# Outcomes of Psychological Therapies for Prisoners With Mental Health Problems: A Systematic Review and Meta-Analysis

**DOI:** 10.1037/ccp0000214

**Published:** 2017-06-01

**Authors:** Isabel A. Yoon, Karen Slade, Seena Fazel

**Affiliations:** 1 Department of Psychiatry, University of Oxford; 2 Division of Psychology, Nottingham Trent University; 3 Department of Psychiatry, University of Oxford, and Oxford Health NHS Foundation Trust, Oxford, United Kingdom

**Keywords:** cognitive behavioral therapy, depression, meta-analysis, mindfulness, prison

## Abstract

***Objective:*** Prisoners worldwide have substantial mental health needs, but the efficacy of psychological therapy in prisons is unknown. We aimed to systematically review psychological therapies with mental health outcomes in prisoners and qualitatively summarize difficulties in conducting randomized clinical trials (RCTs). ***Method:*** We systematically identified RCTs of psychological therapies with mental health outcomes in prisoners (37 studies). Effect sizes were calculated and meta-analyzed. Eligible studies were assessed for quality. Subgroup and metaregression analyses were conducted to examine sources of between-study heterogeneity. Thematic analysis reviewed difficulties in conducting prison RCTs. ***Results:*** In 37 identified studies, psychological therapies showed a medium effect size (0.50, 95% CI [0.34, 0.66]) with high levels of heterogeneity with the most evidence for CBT and mindfulness-based trials. Studies that used no treatment (0.77, 95% CI [0.50, 1.03]) or waitlist controls (0.71, 95% CI [0.43, 1.00]) had larger effect sizes than those that had treatment-as-usual or other psychological therapies as controls (0.21, 95% CI [0.01, 0.41]). Effects were not sustained on follow-up at 3 and 6 months. No differences were found between group and individual therapy, or different treatment types. The use of a fidelity measure was associated with lower effect sizes. Qualitative analysis identified difficulties with follow-up and institutional constraints on scheduling and implementation of trials. ***Conclusions:*** CBT and mindfulness-based therapies are modestly effective in prisoners for depression and anxiety outcomes. In prisons with existing psychological therapies, more evidence is required before additional therapies can be recommended.

## What is the public health significance of this article?

This study suggests that CBT and mindfulness-based therapies are modestly effective in prisoners in treating depression and anxiety symptoms. No clear difference between group and individual-based treatments were found. Additional psychological treatments need stronger evidence before they could be considered in jails and prisons.

It is estimated that 10% to 12% of people in jails and prisons have diagnoses of major depression (Fazel & Seewald, 2012), 4% have psychotic illnesses (Fazel & Seewald, 2012), and the prevalence of posttraumatic stress (Goff, Rose, Rose, & Purves, 2007), anxiety and personality disorders are higher than that of the general population of similar ages (Butler et al., 2006; Trestman, Ford, Zhang, & Wiesbrock, 2007). With more than 10 million people in jails and prisons worldwide (Walmsley, 2013), a substantial burden of psychological morbidity is thus found in prisoners. These mental health problems are risk factors for a range of adverse outcomes in prison and on release including self-harm (Hawton, Linsell, Adeniji, Sariaslan, & Fazel, 2014), suicide (Fazel, Cartwright, Norman-Nott, & Hawton, 2008; Fazel, Grann, Kling, & Hawton, 2011; Haglund et al., 2014; Pratt, Piper, Appleby, Webb, & Shaw, 2006; Rivlin, Hawton, Marzano, & Fazel, 2010; Verona, Hicks, & Patrick, 2005), and violence inside prison (Goncalves et al., 2014) and reoffending in released prisoners (Baillargeon, Binswanger, Penn, Williams, & Murray, 2009; Chang, Larsson, Lichtenstein, & Fazel, 2015; Shaffer et al., 2007). To address this, many countries have introduced specialist mental health services in prisons but these vary considerably within and between countries, including for psychological therapies. Little is known about which treatments are based on good quality evidence, which may not be generalizable from community settings because of the particular challenges of delivering treatment in prisons based on individual characteristics (including comorbidity) and the nature of the environment.

A number of systematic reviews of mental health interventions for prisoners have been published (Bartlett et al., 2015; Fontanarosa, Uhl, Oyesanmi, & Schoelles, 2013; Heckman, Cropsey, & Olds-Davis, 2007; Himelstein, 2011; Kouyoumdjian et al., 2015; Leigh-Hunt & Perry, 2015; Morgan & Flora, 2002; Morgan et al., 2012; Ross, Quayle, Newman, & Tansey, 2013; Shonin, Van Gordon, Slade, & Griffiths, 2013; Sirdifield, Gojkovic, Brooker, & Ferriter, 2009). However, they mostly focus on selected populations and disorders (Leigh-Hunt & Perry, 2015), specific therapies (Shonin et al., 2013) and combine randomized and nonrandomized trials (Bartlett et al., 2015; Morgan et al., 2012). Other reviews have been broader literature reviews that examined different study designs (including theoretical papers, audits, needs assessments, and screening; Sirdifield et al., 2009) or included interventions outside prison (Fontanarosa et al., 2013). One review of English-language studies that covered a broad range of interventions and outcomes using dichotomous diagnoses found a strong effect size (ES = 0.87) but did not explore sources of heterogeneity or compare the outcomes by treatment type (Morgan et al., 2012). Another recent review covered RCTs to improve health during imprisonment and a year after release, but this review covered a wide range of mostly physical health and drug abuse interventions (Kouyoumdjian et al., 2015), did not metaanalyze findings, and used a search strategy that was not optimized for identifying psychological treatments. Thus, previous reviews have been limited in examining the efficacy of psychological therapies by either being too specific or overly broad.

This paper aims to address these gaps by conducting a systematic review and meta-analysis on solely RCTs of psychological therapies of unselected samples of prisoners. For the purposes of this review, prisoners are considered to be presentenced (also known as remand prisoners or detainees) and sentenced individuals in jails and prisons, but not persons in police custody or other forms of administrative detention (such as immigrant detention centers). We sought to compare effect sizes across different types of psychological therapies and examine sources of heterogeneity. In addition, we qualitatively examined the difficulties in implementing RCTs of psychological therapies in prisons in order to make further recommendations for research.

## Method

### Protocol and Registration

The Preferred Reporting Items for Systematic Reviews and Meta-analyses (PRISMA) guidelines were followed (Moher, Liberati, Tetzlaff, & Altman, 2009), and the protocol was prospectively registered in PROSPERO (n.d.; the International prospective register of systematic reviews) to minimize reporting bias through adherence to the initial protocol and to avoid duplication so that researchers can see what systematic reviews are in progress before undertaking their own.

### Search Strategy

PsycINFO, MEDLINE, Global Health, PubMed, CINAHL, National Criminal Justice Reference Service, Scopus, EMBASE, and Cochrane Library were searched from their start dates until May 30, 2015. Additional targeted searches were conducted by hand-searching citations and reference lists of other systematic reviews and articles. Targeted searches on specific authors (identified from previous papers), mindfulness-based therapies, and treatments for psychopathy were conducted separately. We corresponded with authors to clarify data when necessary. Details about keywords are outlined in Appendix A.

### Study Eligibility

Inclusion and exclusion criteria were as follows:

#### Study design

RCTs including pilot studies and cluster-randomized trials were included. Nonrandomized trials (including pretest/posttest comparisons) and case studies were excluded.

#### Population

Prisoners (including juveniles, remand, detainees) were included. Samples not currently in prison (e.g., post-prison release treatments (Sacks, McKendrick, & Hamilton, 2012), people on parole, and in secure hospitals or therapeutic communities outside prisons) were excluded.

#### Interventions

Cognitive behavioral therapy, dialectical behavior therapy, Mindfulness-based Therapy, and other group treatments such as Music Therapy and Art Therapy (including self-help treatments) were included. Studies examining only medication were excluded.

#### Outcomes

Studies that reported psychological improvement measured by standardized instruments at posttreatment and follow-up were included. Outcomes restricted to recidivism or substance use were excluded.

#### Language

Studies in any language including unpublished (e.g., doctorates) reports were considered. Studies that did not provide data to calculate effect sizes were excluded.

Studies treating psychopathy or sociopathy in prisons were not included because none of the identified studies had standardized psychological outcomes.

### Data Extraction and Quality Assessment

In addition to effect sizes, 95% confidence intervals, variance of outcomes, and prespecified study characteristics were recorded. Primary outcome was selected as being the most commonly used psychological assessment in the included study to facilitate comparisons. A second extractor (a consultant psychiatrist with prison experience) extracted data independently, and any disagreements were resolved.

Eligible studies were assessed using the quality checklist used by the National Institute of Health and Care Excellence (NICE; see Appendix B), which assesses internal validity such as the use of adequate concealment method for participant allocation (concealing the allocation sequence from research and clinical staff and participants until permanent assignment of participants into each study group), blinding of subjects and investigators, and intention-to-treat analyses. Overall rating was either: − (few or no criteria fulfilled), + (some fulfilled), or ++ (all or most fulfilled).

### Statistical Analysis

#### Effect size calculation

The standardized mean difference (*d*), 95% confidence intervals, and variance were calculated for each study (Wilson, 2001). For studies with more than one control group, the one that received more therapy was chosen over the waitlist control in order to have a more conservative estimate. For a study that compared two different treatment groups, each treatment group was independently compared with controls. Double-counting of the participants did not apply as no studies reported participants in both intervention groups (Higgins, 2011).

#### Meta-analysis

Given the clinical heterogeneity between studies, random-effects models were conducted. The degree of statistical heterogeneity was assessed using I^2^, which represents the percentage of the observed variation in effect size across studies due to true heterogeneity rather than chance (Higgins & Thompson, 2002) with values of 25%, 50%, and 75% indicating low, moderate, and high levels of heterogeneity, respectively (Higgins, Thompson, Deeks, & Altman, 2003).

Effect sizes were grouped into domains and presented in forest plots. First, the studies were grouped by comparator type: one group of studies included no treatment (including no-contact group) as controls, and another included waitlist as controls. A final group included active treatment controls, such as treatment-as-usual or another form of psychological therapy such as individual supportive therapy, standard prison-based therapeutic community, supportive group therapy (SGT), or attention-matched manualized psychoeducation (Ford, Chang, Levine, & Zhang, 2013; Johnson & Zlotnick, 2012; Messina, Grella, Cartier, & Torres, 2010; Perkins, 1998; Wilson, 1990).

Second, we stratified studies by treatment type: CBT-based, mindfulness-based, trauma-based, and ‘other’ therapies. These categories were chosen so that they included at least 5 studies (the minimum prespecified number of studies). In the CBT-based category, in addition to traditional CBT techniques (Khodayarifard, Shokoohi-Yekta, & Hamot, 2010), therapies using CBT principles (e.g., Seeking Safety [Wolff et al., 2015; Zlotnick, 2002; Zlotnick, Johnson, & Najavits, 2009] and Cognitive Processing Therapy [Ahrens & Rexford, 2002]) were combined. For the mindfulness-based category, meditation was included (Abrams & Siegel, 1978). Trauma-based category included therapies that were trauma-focused and targeted at improving trauma symptoms (Bradley & Follingstad, 2003; Cole, Sarlund-Heinrich, & Brown, 2007; Richards, Beal, Seagal, & Pennebaker, 2000; Valentine & Smith, 2001; Wolff et al., 2015). In “other,” Interpersonal Psychotherapy (Johnson & Zlotnick, 2012), mother–infant attachment based therapy (Sleed, Baradon, & Fonagy, 2013), Gender Responsive Treatment (Messina et al., 2010), art therapy (Gussak, 2007, 2009), music therapy (Chen, Hannibal, & Gold, 2016; Gold et al., 2014), and video pretraining (Hilkey, Wilhelm, & Horne, 1982) were included. Two therapies that combined CBT and mindfulness (Lanza, García, Lamelas, & González-Menéndez, 2014; Messina et al., 2010) were considered “other.”

Test of between-groups heterogeneity of these subgroups by treatment type were conducted using the mixed-effect analysis (Borenstein, Hedges, Higgins, & Rothstein, 2011).

#### Subgroup analyses

Subgroup analyses investigated treatment format (group, individual, or combination) and individual outcomes (e.g., depression).

### Metaregression and Publication Bias

Metaregression analysis was performed to examine sources of heterogeneity on a range of prespecified factors. For the dichotomous version of the gender variable, more than 90% of male was classified as male even when total sample included some females. Because of a large number of U.S.-based studies (*n* = 26) and few studies from each of the other countries included, the variable of country setting was analyzed as U.S. versus rest of the world.

In metaregression, variables in univariate analyses with *p* values of <0.l were included in multivariable models. Multivariable analysis was conducted with all of the variables simultaneously with either the dichotomous or continuous version of each variable to avoid collinearity (Chatterjee & Hadi, 2006). If there were fewer than 10 studies that reported the explanatory variable(s) of interest, metaregression analysis was not performed (Thompson & Higgins, 2002).

To test for publication bias, funnel plot analysis and Egger’s test were performed (Sterne & Egger, 2001; Sterne et al., 2011; Tacconelli, 2010). As an exploratory analysis, the trim and fill analysis (with random-effects model) was also conducted with the total sample and subset of samples (studies with no treatment/waitlist controls) to identify and correct for funnel plot asymmetry attributable to publication bias (Higgins, 2011; Peters, Sutton, Jones, Abrams, & Rushton, 2007). Analyses were performed in STATA-IC 14.

### Qualitative Analysis

For a qualitative analysis on the difficulties of conducting RCTs of psychological therapies in prisons, the discussion sections (and in particular the limitations parts) of included studies were reviewed through a thematic analysis, which identifies key recurrent messages from series of studies (Bearman & Dawson, 2013). The identified factors were organized thematically by the frequency of their appearance in these studies, and those that were mentioned by at least two independent researchers were extracted for the purposes of this synthesis.

## Results

### Main Results

#### Study characteristics

**Figure 1 fig1:**
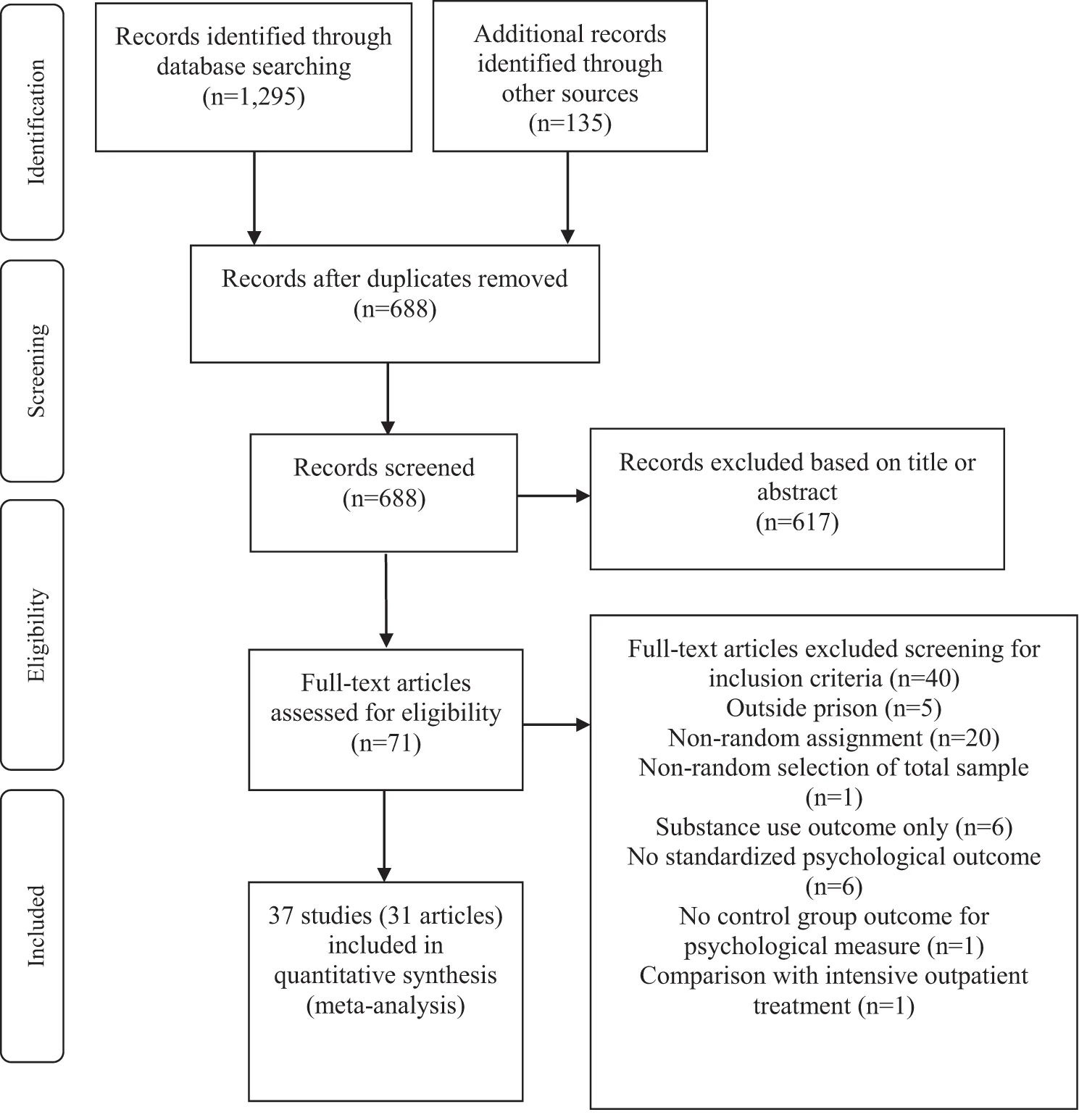
PRISMA flow diagram of search strategy for systematic review and meta-analysis.

We identified 37 studies from 31 publications (see Figure 1) between 1979 and 2015 from 7 different countries (China, India, Iran, Norway, Spain, US, and U.K.). This included 2,761 prisoners, 59% of whom were male. The mean age was 31.8 years (adult prisoners: 34.4 years, juveniles: 16.9 years). All identified studies recruited voluntary participants through informed consent, and none of the studied treatments were mandatory. Sixteen studies had either a specific diagnosis such as PTSD (*n* = 6) and depression (*n* = 2) or specific symptoms in their inclusion criteria (see Appendix C for details of included studies).[Fig fig1]

#### Treatment targets and types of outcome measures

The included RCTs focused on the following primary outcomes: depressed mood (*n* = 20), anxiety (*n* = 21), trauma symptoms (*n* = 10), and overall psychopathology (*n* = 17). Secondary outcomes were somatization (*n* = 9) and hostility/anger (*n* = 11). The most common primary outcome measures reported were: Beck Depression Inventory (BDI; Beck, Ward, Mendelson, Mock, & Erbaugh, 1961), BDI-II (Beck, Steer, & Brown, 1996), global severity index of the Brief Symptom Inventory (BSI; Derogatis & Spencer, 1993), Clinician Administered PTSD Scale (CAPS; Blake et al., 1995), State–Trait Anxiety Inventory (STAI; Spielberger, 1989), Youth Self Report (YSR; Achenbach, 1991), and global severity index of the Symptom Checklist Revised (SCL-90–R; Derogatis & Unger, 2010). All primary outcome measures were validated (see Appendix C for list) apart from an insomnia checklist (Sumter, Monk-Turner, & Turner, 2009).

#### Treatment length and quality rating

Treatment length typically ranged from 10 days to 18 weeks with a mean of 10 weeks. None of the studies were excluded based on quality rating. However, seven studies of the 37 studies met the highest quality rating (Ford et al., 2013; Johnson & Zlotnick, 2012; Messina et al., 2010; Mitchell et al., 2011; Pardini et al., 2014; Sleed et al., 2013; Valentine & Smith, 2001; see Appendix D).

In addition, there were 12 studies with a satisfactory fidelity measure of treatment, 5 with a partial measure, 9 studies without any measure and 11 studies not reporting. Seven studies used double-blinding.

#### Overall effect sizes

**Figure 2 fig2:**
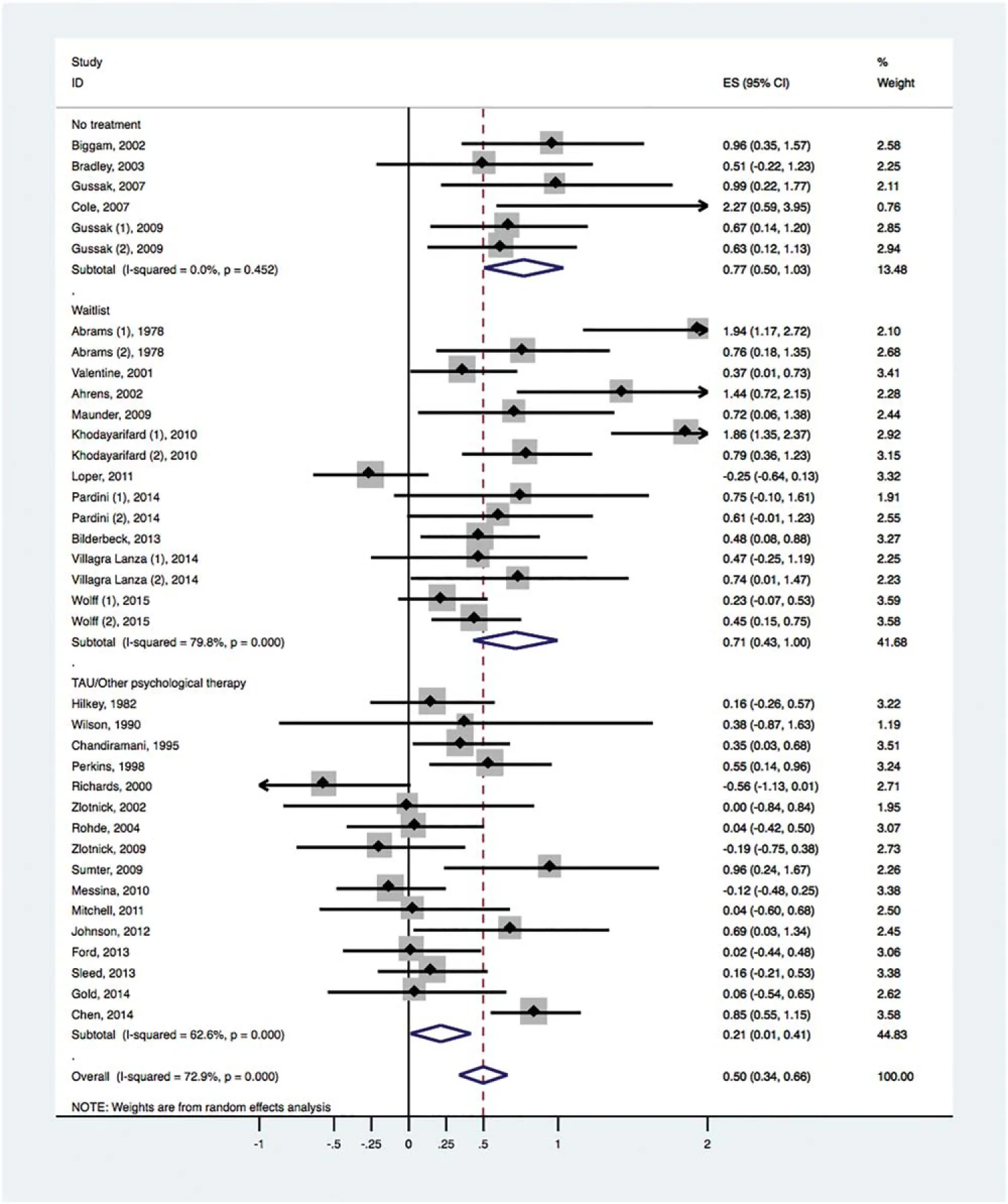
Effect sizes of RCTs for psychological treatments in prisons with mental health outcomes (by comparator type). ES = effect size; TAU = treatment as usual. See the online article for the color version of this figure.

Psychological treatments had a pooled effect size of 0.50 (95% CI [0.34–0.66]) with high levels of heterogeneity (I^2^ = 73%; 95% CI [62%, 80%]). Higher effect sizes were reported in studies with no treatment controls (ES = 0.77; 95% CI [0.50, 1.03]; I^2^ = 0%, 95% CI [0%, 75%]) and waitlist controls (ES = 0.71; 95% CI [0.43, 1.00]; I^2^ = 80%, 95% CI [67%, 87%]) than those with active treatment controls (ES = 0.21; 95% CI [0.01, 0.42]; I^2^ = 63%, 95% CI [36%, 78%]; see Figure 2).[Fig fig2]

#### Specific types of outcomes

**Figure 3 fig3:**
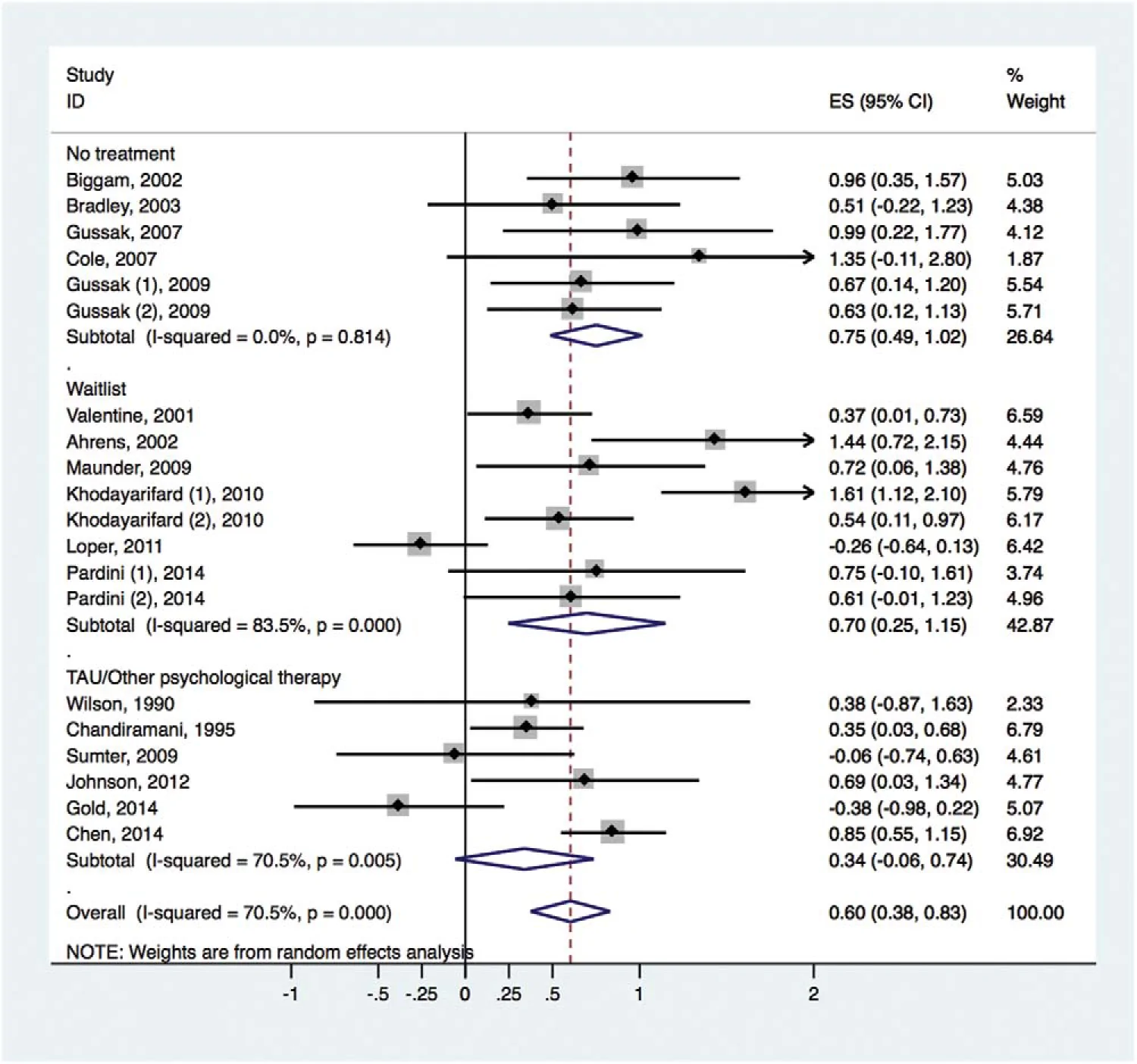
Effect sizes of RCTs of psychological treatments for depression outcomes in prisoners (by comparator type). ES = effect size; TAU = treatment as usual. See the online article for the color version of this figure.

Twenty studies that measured depression outcomes had a pooled effect size of 0.60, 95% CI [0.38, 0.83] with high heterogeneity (I^2^ = 71%, 95% CI [54%, 81%]; see Figure 3). There were higher effect sizes in the trials that used no treatment and waitlist controls.[Fig fig3]

Psychological treatments were effective for other mental health outcomes including anxiety, overall psychopathology, trauma, and anger/hostility but not for somatization (see Table 1).[Table tbl1]

#### Effect sizes at follow-up

Six studies investigated outcomes at 3 months posttreatment, and reported a nonsignificant pooled effect size of 0.29, 95% CI [−0.05, 0.64]; I^2^ = 62%, 95% CI [8%, 84%]. When one study (Wolff et al., 2015) which compared two active treatments was removed, there was little difference (ES = 0.35; 95% CI [0.09, 0.79]). Five studies that reported outcomes at 6 months after treatment found no effect (ES = 0.06; 95% CI [−0.15, 0.26]; I^2^ = 0%, 95% CI [0%, 79%]).

#### Sensitivity analysis

Removal of one outlier (*n* = 9) with a large effect size (*d* = 2.27; Cole et al., 2007) did not materially change the overall effect (ES = 0.48; 95% CI [0.32, 0.65]; I^2^ = 73%, 95% CI [62%, 80%]) nor that of the subgroup with no treatment controls (ES = 0.73; 95% CI [0.46, 1.00]; I^2^ = 0%, 95% CI [0%, 79%]).

#### Secondary analyses

**Figure 4 fig4:**
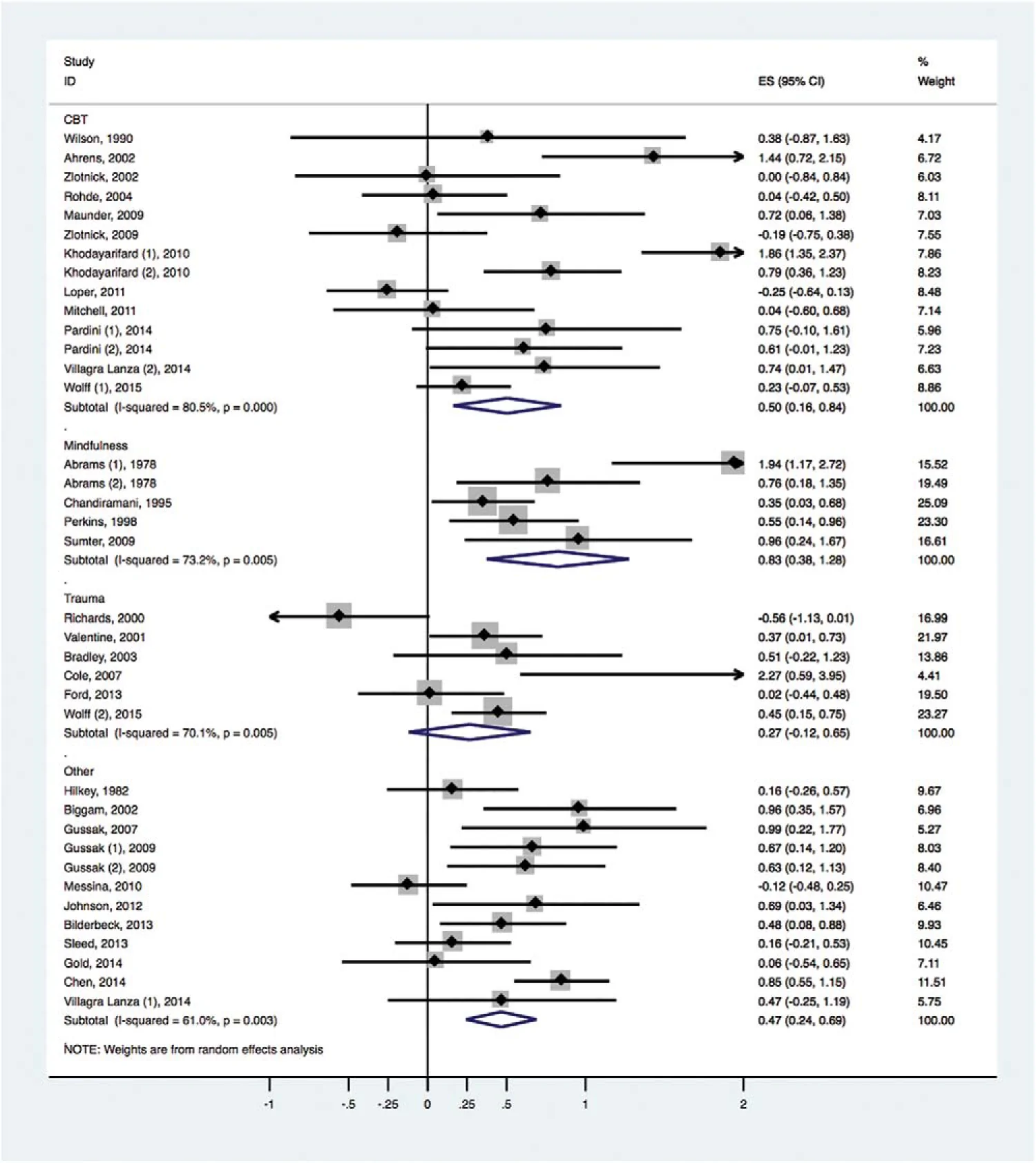
Effect sizes of RCTs from different types of psychological treatment in prisoners. ES = effect size. See the online article for the color version of this figure.

No clear differences were found between group therapy and individual therapy (ES = 0.43; 95% CI [0.26, 0.60] vs. ES = 0.38; 95% CI [0.02, 0.74]), or combination therapy (ES = 0.72; 95% CI [0.25, 1.19]). When the studies were stratified by treatment type, effect sizes did not significantly differ (see Figure 4).[Fig fig4]

### Metaregression Results

#### Univariate metaregression analysis

Higher attrition rates and the use of no treatment/waitlist controls correlated with higher effect sizes (see Table 2).[Table tbl2]

#### Multivariable metaregression analysis

In multivariable models, the fidelity measure was significant, β = −0.86, *SE*(β) = 0.30, *p* = .02. Even when missing data (*n* = 10) were assumed to not use a fidelity measure, the variable remained significant in 32 studies, β = −0.52, *SE*(β) = 0.23, *p* = .03.

#### Metaregression within subgroups

In studies with waitlist controls, sample size (dichotomous) was significant in the univariable analysis (*p* < .05). In the studies with no treatment controls, metaregression analysis was limited because of a small number of studies and high levels of collinearity between variables (e.g., retention rate, sample size). When retention rate, publication year, and sample size were included in multivariable analysis for waitlist control studies, retention rate, β = −0.68, *SE*(β) = 0.25, *p* = .021, and sample size (as a continuous variable: β = −0.006, *SE*(β) = 0.002, *p* = .032; dichotomous variable: β = −0.68, *SE*(β) = 0.21, *p* = .007) remained significant.

#### Difficulties of conducting RCTs in prisons (thematic analysis)

A thematic analysis was conducted on the same 37 studies included in the quantative analysis (Sleed et al., 2013). The main themes identified were posttreatment follow-up and institutional constraints. The most common theme was difficulties with posttreatment follow-up (Chandiramani, Verma, & Dhar, 2000; Cole et al., 2007; Gussak, 2007; Maunder et al., 2009; Perkins, 1998; Valentine & Smith, 2001) because of high rates of release (Chandiramani et al., 2000), rapid turnover of prisoners (Sleed et al., 2013), short duration of stay (Gold et al., 2014), with difficulties in ensuring continuity of care (Mitchell et al., 2011; Wolff et al., 2015).

The second most commonly identified problem was institutional constraints which reflected two main subcategories: constraints on the scheduling of sessions (e.g., scheduling conflicts with other activities (Ford et al., 2013) and ‘lock-downs’ (Messina et al., 2010), high attrition rates (Bradley & Follingstad, 2003; Gussak, 2009; Lanza et al., 2014; Loper & Tuerk, 2011; Sleed et al., 2013; Zlotnick, 2002) partly attributable to scheduling changes (Cole et al., 2007; Loper & Tuerk, 2011) and inmate infractions that restricted enrolment into treatment programs (Loper & Tuerk, 2011). The second subtheme—constraints on the implementation of proposed individual study characteristics—covered a broad range including policies against gathering biological markers or video recording (Bilderbeck, Farias, Brazil, Jakobowitz, & Wikholm, 2013; Cole et al., 2007; Ford et al., 2013) and controlling for changes in the social environment of the prison (Biggam & Power, 2002; Chandiramani et al., 2000; Johnson & Zlotnick, 2012; see Appendix E for additional findings).

#### Publication bias

Funnel plot analysis demonstrated nonsignificant evidence of publication bias with the total set of studies, *t* = 1.83, *SE*(*t*) = 0.90, *p* = .08. There was evidence of publication bias in the subgroups with no treatment controls, *t* = 2.38, *SE*(*t*) = 0.86, *p* = .05, and waitlist controls, *t* = 3.57, *SE*(*t*) = 1.46, *p* = .03; see Appendix F. Studies with TAU/other therapy as controls did not show statistical evidence of publication bias, *t* = −0.96, *SE*(*t*) = 1.30, *p* = .36.

## Discussion

We have reported a systematic review and meta-analysis of RCTs of psychological therapies focused on prisoner mental health outcomes based on 37 studies involving 2,761 prisoners. Although the random-effects pooled effect size was 0.50 (95% CI [0.34, 0.66]), which would represent a medium effect (Cohen, 1977), after limiting RCTs to those with active controls, the effect size was reduced to 0.21 (95% CI [0.01, 0.41]). This pattern was consistent for specific mental health problems, such as depression, where there was the most evidence.

### Implications

There were four main implications. First, this review suggests that RCTs of CBT and mindfulness-based therapies have shown moderate evidence to improve depressive and anxiety symptoms in prisoners where no preexisting treatments are in place, with mindfulness-based therapies possibly demonstrating higher effect sizes. The mechanisms underlying such treatment efficacy need exploration (van der Velden et al., 2015). Second, trauma-based therapies demonstrated limited evidence of effect on trauma symptomology. Although the difference between types of therapy was not statistically significant, both a visual analysis and a subgroup analysis of trauma symptom outcomes were consistently lower than other mental health problems such as depression or anxiety. Improving trauma-based treatments should be prioritized given the high prevalence of PTSD in prisons (4–21%; Goff et al., 2007). Prisoners not only arrive with high levels of existing trauma symptoms, but also are prone to traumatic experiences in prison. Therefore, future research should take into account repeat traumas while in prison in the treatment delivery and assessment of outcomes. In contrast, we reported that trauma-based symptoms were reduced after psychological treatments in prisoners, but this was in trials using all therapeutic approaches, not only trauma-based ones. This suggests that reducing trauma symptoms in prisoners may benefit from improving psychological treatments more widely rather than introducing specific types of therapy. Third, it was difficult to come to conclusions about action-oriented approaches (such as art and music therapy) because of the lack of research and the difficulty in interpreting pooled estimates based on different treatments. These methods are not widely available to prisoners but may provide alternatives for those not interested in current treatments and be more cost-effective (Bilderbeck et al., 2013), partly because they are more accessible and less stigmatizing for male prisoners than other psychosocial treatments (Byrne, 2000). A final implication is based on the finding that participation type (group vs. individual) did not significantly differ, which suggests that group therapies could be considered as a baseline psychological intervention if resources are limited—although these will not be appropriate for acute illnesses. Caution is warranted in interpreting the lack of significant difference in format of therapy as there may be other explanations. For example, treatment dosage was different—the average treatment length was 10 weeks for group therapies, 6 weeks for individual therapies, and 12 weeks for combination ones (treatments comprised of weekly or biweekly sessions).

Most of the included trials involved short-term treatment with an average length of 10 weeks. Providing short-term psychological therapies can be efficient, particularly as the review found that the length of treatment did not alter treatment effects. However, as the maintenance of psychological gains was not found at 3 and 6 months, further research is needed to clarify ways to retain short-term gains, and consideration should be given to additional sessions after the ending of a treatment program. In addition, future research should investigate combined individual and group treatments.

Qualitative analysis of difficulties in conducting RCTs in prisons suggested that many obstacles would not be overcome by improving research design as many were secondary to structural factors (such as following up prisoners and scheduling treatments) in conducting research in prisons. The early involvement of the relevant custodial staff and departments in the research design and plans for implementation may address these problems.

We identified shortcomings in trial design in many of the included RCTs. Small samples in particular could be overcome by multicenter trials (Bilderbeck et al., 2013; Chen et al., 2016; Sleed et al., 2013; Wolff et al., 2015). In multisite trials, adherence to the study protocol must be thoroughly checked to ensure that the results are comparable in different sites. In addition, few studies utilized a fidelity measure to ensure consistent quality and delivery of treatment (Bond, Evans, Salyers, Williams, & Kim, 2000). We found that the presence of a fidelity measure was associated with lower effect sizes, possibly because of its association with implementing more stringent study conditions, and thus less prone to bias such as lack of blinding.

Prison populations exhibit high levels of psychopathology but also have elevated levels of comorbidity including personality disorder and substance use. If research and treatment pathways fail to take these comorbidities into account, any treatment approach that focuses on a single diagnostic group may encounter difficulties in identifying and interpreting the true clinical effect or may exclude individuals with notable health and social needs. For example, a pilot scheme in England extending a community service into prison (IAPT) identified that limiting the access for prisoners with more complex presentations excluded high need persons (Forrester, MacLennan, Slade, Brown, & Exworthy, 2014). The provision of more specialist and targeted services should, however, continue to be considered for acute cases and those who do not respond to available treatment approaches. A more joined-up approach between the offending and health pathways may be warranted. Many jurisdictions provide large-scale psychological treatment programs that address offending needs, including in relation to emotional management. These programs have successfully run for decades and although their impact on mental health is uncertain, future research on broader psychological outcomes could be considered.

### Comparisons

Evidence comparing psychological and pharmacological treatments for prisoners is lacking as we did not identify head to head trials. In community settings, however, the overall effect sizes for symptom reduction with antidepressants (0.38, 95% CI [0.34, 0.41]) and antipsychotics (0.51, 95% CI [0.43, 0.59]) are reported to be at comparable levels to the psychological therapies reported here (Leucht, Helfer, Gartlehner, & Davis, 2015). Although treatment effects were not sustained at 3-month and 6-month follow-up for studies that examined longer term outcomes in this review, this contrasts with trials of antidepressants and antipsychotics for acute treatment in the community that appear to be sustained at follow-up (e.g., for antidepressants at 12 weeks: ES = 0.34, 95% CI [0.25, 0.43] and at 24 weeks: ES = 0.34, 95% CI [0.18, 0.50]; for antipsychotics, one review of 12 month outcomes reported risk ratios of 0.40, 95% CI [0.33, 0.49]; Henssler, Kurschus, Franklin, Bschor, & Baethge, 2017; Leucht et al., 2012) although information on longer term effects of medication are limited to observational studies in prisoners (Chang, Lichtenstein, Langstrom, Larsson, & Fazel, 2016), and comparisons will need to take into account the differential adherence patterns between psychotropic medication and psychological treatments. A recent review of mostly CBT, disorder-specific psychotherapies, and psychodynamic approaches reported an effect size of 0.58 (Huhn et al., 2014), similar to our pooled estimate of 0.50. Community-based trials have also found that studies with no treatment/waitlist controls have higher effect sizes than subgroups with more active controls (such as those receiving placebo, treatment as usual, and noneffective therapy; Huhn et al., 2014). This supports the view that active treatment controls are likely to have better posttreatment outcomes than the no treatment/waitlist controls because of placebo or other nonspecific benefits from the intervention offered to the control group. Finally, the current review did not show clear differences in participation format (individual vs. group), similar to community studies (Gaudiano & Miller, 2013).

### Strengths and Limitations

To our knowledge, this is the first comprehensive meta-analysis of all psychological therapies for prisoners. It includes 37 trials, and larger than a previous review of 15 investigations (Morgan et al., 2012), although the latter was focused on prisoners with dichotomous diagnoses. The current review also provides a more conservative estimate of effect (ES = 0.50) than the 2012 review (ES = 0.87), likely because of a larger number of included studies. On the other hand, some limitations need to be considered. Double-blinding is difficult for psychological treatment studies (Huhn et al., 2014). Lack of blinding can favorably bias treatment and imperfect blinding has been a commonly identified issue in other meta-analyses of psychotherapy studies (Gold, Voracek, & Wigram, 2004; Huhn et al., 2014; Sensky, 2005). In addition, there were 8 studies that did not employ intention-to-treat (ITT) analyses (ES = 0.58, 95% CI [0.14, 1.01]), which might favorably bias the treatment group if noncompleters report lower treatment effects, and these were not different to those that used ITT analyses (ES = 0.46, 95% CI [0.29, 0.63]. A further related limitation were the analytic strategies employed. Apart from one trial (Johnson & Zlotnick, 2012), studies did not use analysis of covariance (ANCOVA) when reporting posttreatment outcomes; using pretreatment scores as a covariate in comparing posttreatment scores would yield a more precise effect size estimate (Higgins, 2011). Apart from four investigations (Ford et al., 2013; Wolff et al., 2015; Zlotnick, 2002; Zlotnick, Johnson, & Najavits, 2009), studies included in the review relied on self-report measures for outcomes (Ahrens & Rexford, 2002; Bradley & Follingstad, 2003; Loper & Tuerk, 2011; Rohde, Jorgensen, Seeley, & Mace, 2004; Sumter et al., 2009; Wilson, 1990). However, these are appropriate for many psychological trials; clinical interviews that only check for presence or absence of a formal diagnosis may not be sensitive to treatment change, and many trials did not require a baseline diagnosis. Nevertheless, some triangulation of outcomes (with clinical and possible biological markers) should be considered in future work. Furthermore, outcomes of specific disorders were not examined as a subgroup analysis in this review because of the limited number of studies that required participants to have a clinical diagnosis. Future work could consider recruiting prisoners with certain diagnoses, particularly severe mental disorders that are overrepresented in custodial populations and whose outcomes are worse than other prisoners. The shortage of empirically tested treatments targeting specific psychological diagnoses in prisoners seems to be largely a result of structural factors such as the institutional constraints of prison settings discussed in the thematic analysis in this review (see also Appendix E). However, the fundamental purpose of prisons is not the care and treatment of those with severe mental illness and the emphasis in many jurisdictions is on transferring them to secure hospitals in order to access the full range of appropriate care and treatment within an explicitly therapeutic environment. The interventions that prisoners may access in secure hospitals were not included in the review.

We reported high levels of heterogeneity and our overall effect size should be interpreted with caution. High levels of heterogeneity are not unusual for meta-analyses of RCTs, and partly reflect the diverse populations being studied (Higgins et al., 2003). We addressed this partly by conducting a number of subgroup analyses (by comparator, treatment type, and outcome) and multivariable metaregression on a range of prespecified characteristics. For example, metaregression analyses indicated that higher attrition rates were correlated with higher effect sizes (both univariately in all studies and also in a multivariable analysis for waitlist control studies). In studies that did not complete the intention-to-treat (ITT) analysis, one explanation is that participants who drop out do not complete all the treatment components, and are therefore less likely to benefit. It may also be in part attributed to other factors that have been shown to correlate with treatment dropouts such as format of treatment delivery (e.g., in-person v. self-guided) or number of sessions (Fernandez, Salem, Swift, & Ramtahal, 2015). Furthermore, the finding that retention rate and sample size were significantly associated with between-study heterogeneity in waitlist control studies but not in active treatment controls supports the view that the contribution of such factors is not as strong as it is in better designed studies. In addition, heterogeneity was not high in some of these subgroups such as trials with no treatment controls and those with trauma outcomes. Subgroup and metaregression analyses are potentially informative as they identified some consistent explanations for the variations between studies, which can be used to conduct and interpret future treatment trials in prisoners. However, for some subgroups such as ‘other’ therapies that included a wide range of treatments, clinical heterogeneity means that the pooled effect size should be interpreted with considerable caution. Other limitations of the review include that the metaregression was based on study characteristics that were reported, and there will be other explanations that we were unable to test, such as environmental factors (prison-related conditions, attitudes of correctional staff and other prisoners). The alternative—a systematic review without a meta-analysis—was considered and the information in this review allows for groups of similarly conducted studies to be reviewed. Moreover, we incorporated a qualitative analysis of the barriers to psychological trials in prisons. At the same time, as we have conducted two complementary analyses of heterogeneity, this review is more than simply a presentation of pooled estimates.

In addition, another limitation is that we examined outcomes using continuous symptom scores rather than categorical diagnoses, which meant they were more sensitive to change, and included prisoners without diagnoses at baseline. The alternative—to investigate changes to diagnoses—may be easier to interpret and assist in planning services, but was not feasible due to the lack of relevant studies, and future studies could consider including both continuous and categorical outcomes.

### Conclusion

We found that psychological therapies for mental health outcomes in prisoners were modestly effective when there are no existing psychological treatment programs. However, effects were weaker when active treatment controls and a fidelity measure were used in trials. Whether this level of evidence is sufficiently strong for the introduction of such therapies in prison requires careful review and consideration of other factors including cost-effectiveness.

## Figures and Tables

**Table 1 tbl1:** Effect Sizes of RCTs for Psychological Treatments in Prisoners for Other Reported Mental Health Problems

Type of outcome	Number of studies	ES	95% CI	I^2^	95% CI
Anxiety	21	.56	[.31–.82]	82%	74%–88%
Overall psychopathology	17	.32	[.05–.59]	81%	70%–88%
Trauma	10	.35	[.14–.56]	44%	0%–73%
Somatization	9	.30	[−.24–.83]	89%	82%–94%
Hostility/anger	11	.42	[.13–.71]	69%	43%–84%
*Note*. ES = Effect size.					

**Table 2 tbl2:** Findings on Univariate Meta-Regression of Factors Associated With Between-Study Variation in RCTs of Psychological Treatments in Prisons

Variable	β	*SE* (β)	*p*
Gender	.27	.18	.15
Mean age (continuous)	.01	.01	.45
Age group (adult vs. adolescent)	−.09	.30	.77
Year of study (continuous)	−.01	.01	.38
Country (USA vs. rest of the world)	−.25	.19	.20
Retention rate			
Continuous	−1.55	.62	.02*
Low (≤80%) vs. high (>80%)	−.41	.18	.03*
Sample size			
Continuous	−.002	.001	.16
≤100 vs. >100	−.33	.18	.08
Diagnosis (required vs. not required)	−.16	.18	.37
Study quality (high vs. medium)	−.34	.22	.13
Fidelity measure	−.34	.23	.15
Treatment length	.01	.02	.38
Control group (no TR/waitlist vs. TAU/other therapy)	−.52	.16	<.01**
* *p* < .05. ** *p* < .01.			

## A Search Keywords

Psychological disorders keywords: mental*, psych*, disorder*, depress*, schizo*

Prison setting keywords: prison*, inmate*, jail*, penal, correctional, incarcerated

Intervention keywords: cognitive behavioral/ behavioral therapy, CBT, group therap*, intervention*, treatment*, therapeutic communit*

Study design keywords: RCT*, controlled trial*, randomi#ed controlled trial*, controlled clinical trial*, randomi#ed trial*, randomi#ed clinical trial*, trial*

Additional targeted search for psychological therapies that may have been missed by other search terms were performed with the keywords: therap* and psychotherapy.

## B RCT Quality Checklist Used for Quality Appraisal

**Table:**

Section 1: Internal validity		
1.1	The study addresses an appropriate and clearly focused question.	Well covered Not addressed
		Adequately addressed Not reported
		Poorly addressed Not applicable
1.2	The assignment of subjects to treatment groups is randomized.	Well covered Not addressed
		Adequately addressed Not reported
		Poorly addressed Not applicable
1.3	An adequate concealment method is used.	Well covered Not addressed
		Adequately addressed Not reported
		Poorly addressed Not applicable
1.4	Subjects and investigators are kept ‘blind’ about treatment allocation.	Well covered Not addressed
		Adequately addressed Not reported
		Poorly addressed Not applicable
1.5	The treatment and control groups are similar at the start of the trial.	Well covered Not addressed
		Adequately addressed Not reported
		Poorly addressed Not applicable
1.6	The only difference between groups is the treatment under investigation.	Well covered Not addressed
		Adequately addressed Not reported
		Poorly addressed Not applicable
1.7	All relevant outcomes are measured in a standard, valid and reliable way.	Well covered Not addressed
		Adequately addressed Not reported
		Poorly addressed Not applicable
1.8	What percentage of the individuals or clusters recruited into each treatment arm of the study dropped out before the study was completed?	Well covered Not addressed
		Adequately addressed Not reported
		Poorly addressed Not applicable
1.9	All the subjects are analyzed in the groups to which they were randomly allocated (often referred to as intention-to-treat analysis).	Well covered Not addressed
		Adequately addressed Not reported
		Poorly addressed Not applicable
1.10	Where the study is carried out at more than one site, results are comparable for all sites.	Well covered Not addressed
		Adequately addressed Not reported
		Poorly addressed Not applicable
Section 2. Overall assessment of the study		
2.1	How well was the study done to minimize bias?	Well covered Not addressed
	Code ++, + or −−	Adequately addressed Not reported
		Poorly addressed Not applicable

## C Study Characteristics of Included Studies

**Table:**

Author & year	Location	*N*	% Male	Age (mean)	Diagnosis	Treatment type	Treatment length	Primary outcome	Secondary outcome measures
Abrams & Siegal (1), 1978	USA, CA Folsom State Prison	55	100%	—	Not required	Transcendental Meditation	14 weeks, weekly	STAI – State Anxiety	STAI − Trait Anxiety, Buss-Durkee Hostility Inventory (BDHI)
Abrams & Siegal (2), 1978	USA, CA Folsom State Prison	49	100%	—	Not required	Transcendental Meditation	14 weeks, weekly	STAI – State Anxiety	STAI − Trait, Buss-Durkee Hostility Inventory (BDHI)
Ahrens & Rexford, 2002	USA, youth correctional facility	38	100%	16.4	PTSD	CPT (Cognitive Processing Therapy)	8 weeks: eight 60-min sessions	BDI	PTSD Symptom Scale-Self Report (PSS-SR)
Biggam & Power, 2002	Scotland, Young Offenders Institution	46	100%	19.3	Not required	Problem solving group-based intervention	Five 90-min sessions	HADS Depression	HADS Anxiety
Bilderbeck, 2013	UK, 7 prisons	167	93%	36.7	Not required	Yoga	10 weeks	BSI	Perceived Stress Scale (PSS)
Bradley & Follingstad, 2003	USA, south-eastern state medium-security prison	31	0%	36.7	Trauma symptom and/or Depression	Trauma treatment group therapy (DBT-based)	18 sessions, 9–2.5 hrs, 9-writing	BDI	Trauma Symptom Inventory (TSI), TSI − anger and irritability
Chandiramani, 1995	India, Tihar Jail	150	100%	—	Not required	Vipassana Retreats (meditation-based)	10 days	BDI	Hamilton Anxiety Rating Scale
Chen, 2015	China, Beijing, adult male prison	194	100%	35.5	Anxiety level ≥ 49 on STAI or depression level ≥ 14 on BDI	Group Music Therapy	20 session: 90-mi, twice weekly	BDI	STAI
Cole, 2007	USA, Washington Correction Center for Women	9	0%	31	Childhood Sexual Abuse	Time-Limited Trauma-focused Therapy Group	8 weeks: 16 sessions, biweekly, 2.5 hrs	SCL-90-R (Global Severity Index)	Trauma symptom inventory
Ford, 2013	USA, York Correctional Institution (Connecticut state prison)	72	0%	34.6	Full/partial PTSD	Trauma Affect Regulation: Guide for Education and Therapy (TARGET)	Twelve 75-minute sessions	Clinician Administered PTSD Scale (CAPS) − Total PTSD Symptoms	Trauma Symptom Inventory (TSI), Clinical Outcomes in Routine Evaluation-Outcome Measure (CORE-OM)
Gold, 2014	Norway, Bergen Prison	113	100%	31.38	Not required	Music Therapy	4 sessions median (5.27 sessions average)	STAI − State (State Anxiety)	STAI-Trait, HADS-A (Anxiety), HADS-D (Depression)
Gussak (1), 2009	USA, Florida maximum security prison	60	100%	22–51	Not required	Art Therapy	15 weeks, weekly	BDI-II	—
Gussak (2), 2009	USA, Florida medium-security prison	96	0%	20–51	Not required	Art Therapy	15 weeks, weekly	BDI-II	—
Gussak, 2007	USA, Florida, medium- to maximum security prisons	29	100%	21–59	Not required; but many had Axis I Disorders	Art Therapy	8 weeks: weekly group session	BDI-II	—
Hilkey et al., 1982	USA, Indiana medium-security Federal Penitentiary	89	100%	28.1	Not required	Video pre-training on Group psychotherapy	8 weeks: weekly 90min (30-min pre-training)	STAI—State Anxiety	—
Johnson & Zlotnick, 2012	USA, Rhode Island State Prison	38	0%	35	MDD & SUD	Interpersonal Psychotherapy	8 weeks	Hamilton Rating Scale for Depression (HRSD)	—
Khodayarifard (1), 2010	Iran, Rajaee Shahr Prison	86	100%	48.2	Not required	CBT (Combined)	Avg. (8) 1-hr weekly group + (8) 1-hr weekly individual sessions	SCL-90-R (Global Severity Index)	SCL-90-R-somatization, hostility
Khodayarifard (2), 2010	Iran, Rajaee Shahr Prison (Tehran province)	88	100%	48.2	Not required	CBT (Individual)	16 weeks: 2-hr weekly	SCL-90-R (Global Severity Index)	SCL-90-R-somatization, hostility
Loper & Tuerk, 2011	USA, State prison	106	0%	33	Not required	Parenting Intervention	8 sessions	BSI—Global Severity Index	BSI-somatic, hostility subscores
Maunder, 2009	UK, North England, HMP Acklington	38	100%	35.2	Symptoms of Anxiety, Depression	Self-help materials	4 weeks	Hospital Anxiety and Depression Scale (HADS)	BSI
Messina, 2010	USA, CA Valley State Prison for Women	115	0%	36	Not required	Gender-responsive treatment (GRT)	17 sessions of *Helping Women Recover* + 11 sessions of *Beyond Trauma*	ASI–psychological composite score	ASI—other subscores
Mitchell, 2011	USA, Secure Children’s Homes, Young Offender Institution	40	100%	15.6	Not required but included diagnosed sample	CBT	9.1 sessions mean-weekly session	Youth Self Report (YSR) − Total behavioral & emotional functioning	YSR-externalizing
Pardini (1), 2014	USA, Tuscaloosa, Alabama metropolitan jail	37	78%	29.4	Depression (BDI score ≥ 10)	Bibliotherapy	4 weeks	BDI	SCL-90-R (Global Severity Index), SCL − somatization, hostility subscores
Pardini (2), 2014	USA, Alabama, max security prison	42	100%	32.7	Depression (CES-D score ≥ 10)	Bibliotherapy	4 weeks	BDI-II	HRSD, BSI total
Perkins, 1998	USA, Florida, Tallahassee, Federal Correctional Institution	143	0%	33.9	Not required	Meditation based program	8 weeks: 2hr/wk.	SCL-90-R (Global Severity Index)	Coping Resources Inventory for Stress (CRIS)–physical health subscore, State-Trait Anger Expression Inventory (STAXI)
Richards, 2000	USA, Midwestern max-security psychiatric prison	98	100%	34.5	>1 DSM-III-R diagnosis	Writing about trauma	3 days: 20 min/day	Cognitive Somatic Anxiety Questionnaire (CSAQ)	Pennebaker Inventory of Limbic Languidness (PILL)
Rohde, 2004	USA, Oak Creek Youth Correctional Facility	76	100%	16.3	Not required	Coping Course	8 weeks: 2 times/wk.	YSR—internalizing score	YSR—externalizing score
Sleed, 2013	UK, MBU (Mother Baby Units) in Prisons	153	0%	—	Not required	New Beginnings (Attached-based group intervention)	4 weeks: (8 sessions) 2 session of 2hr/day, 1day/wk.	Center for Epidemiologic Studies Depression Scale (CES-D)	—
Sumter, 2009	USA, Tidewater Detention Center, Chesapeake, Virginia	33	0%	—	Not required	Meditation	7 weeks, 2.5 hr/wk.	Sleeping difficulties—symptom	Emotions – Throw/hit
Valentine & Smith, 2001	USA, Florida Federal Prison	123	0%	32.8	Not required	Traumatic Incident Reduction Therapy	3–4 hours	BDI	Clinical Anxiety Scale (CAS), PTSD Symptom Scale (PSS)
Villagra Lanza (1), 2014	Spain, Module 10, Prison of Villabona (Asturias)	31	0%	32	SUD	ACT (Acceptance and Commitment Therapy)	16 weeks: weekly 90-min sessions	Anxiety Sensitivity Index (ASI) − Total	ASI − somatic subscore
Villagra Lanza (2), 2014	Spain, Villabona Prison	50	0%	33.2	SUD	CBT	16 weeks: weekly 90-min sessions	Anxiety Sensitivity Index (ASI)–Total	ASI-somatic subscore
Wilson, 1990	USA, large maximum-security prison	10	100%	33.1	Depression	Group cognitive therapy	Fourteen 90-min sessions (21 hrs)	BDI	—
Wolff (1) et al., 2015	USA, Pennsylvania, high security prison	173	100%	42.5	Not required	Seeking Safety	14 weeks	CAPS score (mean)	BSI – Global Severity Index (mean)
Wolff (2) et al., 2015	USA, Pennsylvania, high security prison	175	100%	—	Not required	Male-Trauma Recovery Empowerment Model (M-TREM)	14 weeks	CAPS score (mean)	BSI – Global Severity Index (mean)
Zlotnick, 2002	USA, Rhode Island Adult Correctional Institution	28	0%	30.9	SUD & PTSD	Seeking Safety	12 weeks: 90 min- twice/wk.	Clinician Administered PTSD Scale (CAPS-I)	Addiction Severity Index (ASI)
Zlotnick, 2009	USA, minimum security wing of women’s prison	49	0%	34.6	PTSD & SUD	Seeking Safety	6–8 wks.: 90min, 3 sessions per/week; after release 60-min weekly booster for 12 weeks	CAPS-I	BSI
*Note*. Items not recorded in the study are left blank. Secondary outcome measures include only outcome measures that were examined in the analyses. *N* = sample size; wk. = week; hr = hour. For studies that did not provide mean age but report age range of the sample, the range was recorded.									

## D Quality Rating of Included Studies (Using Quality Checklist From Appendix B)

**Table:**

Author & year	1.1 question	1.2 random	3 concealment	4 double-blind	5 similar group	6 controlled	7 standard outcome measure	8 attrition rate	9 ITT (Intention-to-Treat analysis)	10 multiple site equiv.	2.1 overall
Abrams & Siegal (1), 1978	1	1	N/A	N/A	1	1	1	27%	1	1	+
Abrams & Siegal (2), 1978	1	1	N/A	N/A	1	1	1	17%	1	1	+
Ahrens & Rexford, 2002	1	1	N/A	N/A	2	1	1	0% (not explicitly stated)	1	1	+
Biggam & Power, 2002	1	1	2	2	1	2	1	41%	1	1	+
Bilderbeck, 2013	1	1	N/A	N/A	1	1	1	31%	N/A	1	+
Bradley & Follingstad, 2003	1	1	N/A	N/A	1	1	1	37%	1	1	+
Chandiramani, 1995	1	2	N/A	N/A	2–more illiterate in experimental group	2	1	0% (not explicitly stated)	1	1	+
Chen, 2015	1	1	1	N/A	1	1	1	8%	1	1	+
Cole, 2007	1	1	N/A	N/A	1	3–higher baselines in expr (effect on small sample size)	1	31%	1	1	+
Ford, 2013	1	1	1	1	1	1	1	10%	2	1	++
Gold, 2014	1	1	N/A	N/A	1	1	1	39%	1	1	+
Gussak (1), 2009	1	1	N/A	N/A	1	1	1	48%	N/A	1	+
Gussak (2), 2009	1	1	N/A	N/A	1	1	1	24%	N/A	1	+
Gussak, 2007a	1	1	2	2	3–at beginning of experiment: BDI score higher in expr group)	3	1	41%	1	1	+
Hilkey et al., 1982	1	1	N/A	2	2	1	1	0%	1	1	+
Johnson & Zlotnick, 2012	1	1	1	1	1	1	1	11%	1	1	++
Khodayarifard (1), 2010	1	1	N/A	N/A	1	1	1	28%	N/A	1	+
Khodayarifard (2), 2010	1	1	N/A	N/A	1	1	1	27%	N/A	1	+
Loper & Tuerk, 2011	1	1	N/A	N/A	1	2	1	18%	1	1	+
Maunder, 2009	1	1	1	1–blind researcher	1	1	1	50%	1	1–pilot study	+
Messina, 2010	1	1	1	N/A	1	1	1	18%	1	1	++
Mitchell, 2011	1	1	1	1	1	1	1	5%	1	1	++
Pardini (1), 2014	1	1	1	N/A	1	1	1	38%	1	1–pilot study	+
Pardini (2), 2014	1	1	1	1–blind researcher	1	1	1	17%	1	1	++
Perkins, 1998	1	1	N/A	N/A	1	1	1	10%: 15 drop out; other attrition due to transfers, WRIT, release	N/A	1	+
Richards, 2000	1	1	N/A	N/A	1	1	1	7%	N/A	1	+
Rohde, 2004	1	1	N/A	N/A	2	2	1	0%	1	1	+
Sleed, 2013	1	1	1	1	2	1	1	29%	1	1	++
Sumter, 2009	1	1	N/A	N/A	1	1	3	0% (not explicitly stated)	1	1	+
Valentine & Smith, 2001	1	1	2	N/A	1	1	1	17%	1	1	++
Villagra Lanza (1), 2014	1	1	N/A	N	2	2	1	19% at follow-up; 0% at post-test	1	1	+
Villagra Lanza (2), 2014	1	1	N/A	N/A	1	1	1	3%	1	1	+
Wilson, 1990	1	1	2	N/A–same therapist for all conditions	1	1	1	0%	1	1	+
Wolff (1), 2015	1	2–random & preference groups; no sig. difference between two groups	N/A	N/A	1	2	1	13%	1	1	+
Wolff (2), 2015	1	2–random & preference groups; no sig. difference between two groups	N/A	N/A	1	2	1	10%	1	1	+
Zlotnick, 2002	1	1	N/A	N/A	1	1	1	0%	N/A	1	+
Zlotnick, 2009	1	1	N/A	N/A	1	1	1	10%	1	1	+
*Note*. Full description of quality checklist questions can be found in Appendix B. Abbreviations: equiv. = equivalent; sig. = significant; expr = experimental. Key for rating: 1 = Well covered; 2 = Adequately addressed; 3 = Poorly addressed; N/A = Not addressed/not applicable; ++ = all or most of the criteria fulfilled (unfulfilled criteria very unlikely to alter conclusions of study); + = some of the criteria fulfilled (unfulfilled criteria unlikely to alter conclusions of study).											

## E Additional Qualitative Research Findings

### Theme 1: Sample Size

Limitations included small sample sizes (Cole, Sarlund-Heinrich, & Brown, 2007; Ford et al., 2013; Gussak, 2007; Johnson & Zlotnick, 2012; Lanza et al., 2014; Perkins, 1998; Wilson, 1990; Zlotnick, 2002) as a result of difficulty in participant recruitment (Cole et al., 2007; Pardini et al., 2014), and in one juvenile study, parental non-consent (Mitchell et al., 2011).

### Theme 2: Follow-Up Difficulties

One author commented that a combination of small sample, volunteer participants, and time constraints prevented the collection of post-test follow-up measures (Cole et al., 2007). Non-attendance after release (Mitchell et al., 2011) and short treatment period (Perkins, 1998) were related limitations as a longer period may have been needed to better capture the treatment effects.

### Theme 3: Environmental Constraints

Control group participants were more likely to drop out of a study (Zlotnick, 2002), and one author commented that establishment disruptiveness may have increased the attrition rate (Gussak, 2009). Potential contamination of treatment and control conditions was suspected due to the closed communal setting of the prison wing (Zlotnick, Johnson, & Najavits, 2009). Reliance on self-report measures was also repeatedly identified as a problem (Ahrens & Rexford, 2002; Bradley & Follingstad, 2003; Loper & Tuerk, 2011; Rohde et al., 2004; Sumter, Monk-Turner, & Turner, 2009; Wilson, 1990). One study mentioned the possibility that “inmates wanted to ‘fake good’ in order to be considered for parole or placement evaluation,” even though the authors believed it unlikely (Bradley & Follingstad, 2003). Not being able to blind the participants to treatment condition (Rohde et al., 2004) was a further constraint on the study validity. Finally, in some cases, the prison clinical staff were not expected to be able to re-produce the conditions of safety and confidentiality provided by the treatment facilitators in the study (Wolff et al., 2015).

## F Contoured Funnel Plot With Waitlist Controls

Red axis in the center indicates observed pooled effect estimate.
